# Coagulation potential of concomitant factor VIII administration in people with hemophilia A receiving emicizumab prophylaxis (CAGUYAMA study): a multicenter, open-label, nonrandomized clinical trial

**DOI:** 10.1016/j.rpth.2026.106849

**Published:** 2026-07-17

**Authors:** Masahiro Takeyama, Kenichi Ogiwara, Naoki Ozu, Kana Sasai, Masato Kasahara, Yasuko Furuichi, Toshio Takase, Iyou Nakagawa, Katsuyuki Fukutake, Akira Ishiguro, Kagehiro Amano, Eisuke Adachi, Kumiko Ono, Chiai Nagae, Atsuki Yamashita, Satoshi Higasa, Teruhisa Fujii, Masanori Matsumoto, Midori Shima, Keiji Nogami

**Affiliations:** 1 Department of Pediatrics, Nara Medical University, Nara, Japan; 2 Division of Hemophilia, National Hospital Organization, Osaka National Hospital, Osaka, Japan; 3 Institute for Clinical and Translational Science, Nara Medical University Hospital, Nara, Japan; 4 Division of Pediatrics, Higashiosaka City Medical Center, Osaka, Japan; 5 Division of Pediatrics, Osaka Gyoumeikan Hospital, Osaka, Japan; 6 Division of Pediatrics, Japan Community Health Care Organization Hoshigaoka Medical Center, Osaka, Japan; 7 Division of Blood Coagulation, Ogikubo Hospital, Tokyo, Japan; 8 Center for Postgraduate Education and Training, National Center for Child Health and Development, Tokyo, Japan; 9 Department of Laboratory Medicine, Tokyo Medical University, Tokyo, Japan; 10 Division of Infectious Diseases and Applied Immunology, The Institute of Medical Science, The University of Tokyo, Tokyo, Japan; 11 Division of Joint Surgery, The Institute of Medical Science, The University of Tokyo, Tokyo, Japan; 12 Department of Pediatrics, St. Marianna University School of Medicine, Kawasaki, Japan; 13 Department of Pediatrics, Yokohama City Seibu Hospital, St. Marianna University School of Medicine, Yokohama, Japan; 14 Department of Hematology, Hyogo College of Medicine, Nishinomiya, Japan; 15 Division of Transfusion Medicine and Hemophilia Treatment Center, Hiroshima University Hospital, Hiroshima, Japan; 16 Department of Blood Transfusion Medicine, Nara Medical University, Nara, Japan; 17 The Center of Thrombosis and Hemostasis, Nara Medical University, Nara, Japan

**Keywords:** antibodies, bispecific, blood coagulation tests, emicizumab, factor VIII, hemophilia A

## Abstract

**Background:**

Emicizumab prophylaxis reduces bleeding in people with hemophilia A. However, factor (F)VIII is still required to manage breakthrough bleeding and for surgical procedures. The optimal additional FVIII dose during such events remains unclear because of emicizumab’s baseline hemostatic activity.

**Objectives:**

To assess FVIII-induced changes in global coagulation potential in people with hemophilia A without inhibitors treated with emicizumab and to inform FVIII dosing during prophylaxis.

**Methods:**

The multicenter “Coagulation potential of concomitant factor VIII administration in people with hemophilia A receiving emicizumab prophylaxis” study enrolled 100 people with hemophilia A (aged ≥4 years) receiving emicizumab at 13 centers in Japan. For eligible bleeding or surgical events, FVIII (standard or extended half-life) was administered at a target dose of 30 IU/kg (allowable range, 25–35 IU/kg). Paired blood samples were obtained pre- and post-FVIII administration. The primary endpoint was the change in clot waveform analysis-adjusted maximum coagulation rate, expressed as IMCR% relative to pooled normal plasma and untreated severe hemophilia A plasma. Secondary endpoints included peak thrombin in the thrombin generation assay, rotational thromboelastometry, clinical hemostasis, and safety. Anti-emicizumab antibodies were used *in vitro* to isolate the effects of FVIII.

**Results:**

Thirty-two FVIII-treated events in 24 people with hemophilia A (15 bleeding events and 17 surgical events) were analyzed. The clot waveform analysis-adjusted maximum coagulation rate increased from 39.4% to 92.1% post-FVIII and from 3.2% to 78.6% under anti-emicizumab conditions. Peak thrombin in the thrombin generation assay increased from 249 to 348 nM (IMCR%: from 55.4% to 88.0%) and from 6.4% to 74.6% under anti-emicizumab conditions. All events achieved effective hemostasis. No thromboembolic events, thrombotic microangiopathy, or hypersensitivity reactions were reported.

**Conclusion:**

FVIII administered at a target dose of 30 IU/kg (allowable range, 25–35 IU/kg) improved global coagulation parameters and achieved effective hemostasis without thrombotic complications, warranting evaluation as part of emicizumab prophylaxis.

## Essentials

• Emicizumab (Emi) provides baseline hemostatic activity, but additional FVIII may be required for breakthrough bleeding and procedures.
• In the CAGUYAMA study, people with hemophilia A treated with Emi received FVIII at a target dose of 30 IU/kg (allowable range, 25–35 IU/kg) across 32 clinical events.
• Global coagulation assays revealed substantial additive effects of FVIII beyond the baseline activity of Emi.
• FVIII at a target dose of 30 IU/kg (allowable range, 25–35 IU/kg) was associated with effective hemostasis and no observed thrombotic complications across the evaluated events, and CWA was particularly sensitive to Emi-associated coagulation changes.

## 1 Introduction

Emicizumab is a humanized bispecific monoclonal antibody that bridges activated factor (F)IX and FX, mimicking the cofactor function of activated FVIII in the tenase complex [1]. Across all phase 1–3 clinical trials of emicizumab prophylaxis markedly reduced bleeding rates in people with hemophilia A (HA), with and without FVIII inhibitors, demonstrating a favorable safety profile and the convenience of subcutaneous administration [[2], [3], [4], [5], [6], [7], [8]].

Based on certain global coagulation assays, the hemostatic activity of emicizumab roughly corresponds to that observed in mild HA, approximating a FVIII activity level of 10% to 20% [9,10], a finding corroborated by subsequent clinical and comparative studies [11]. Therefore, in cases of breakthrough bleeding or surgical interventions, additional hemostatic treatment is usually required.

In Japan, clinical practice has generally followed conventional FVIII dosing protocols for breakthrough bleeding or surgeries in people with HA without inhibitors, without specifically accounting for the ongoing hemostatic contribution of emicizumab prophylaxis. For example, in major orthopedic procedures such as total knee arthroplasty, the traditional goal has been to achieve a perioperative FVIII activity of approximately 100% (≈50 U/kg). However, this approach may overlook the additive baseline protection provided by emicizumab prophylaxis, and the optimal FVIII dosing strategy in this setting remains to be established.

Recent consensus recommendations have highlighted the clinical challenges in optimizing FVIII dosing during emicizumab prophylaxis in people with HA without inhibitors, citing the dual risks of thrombotic complications from overdosing and insufficient hemostasis from underdosing [12]. Furthermore, optimizing the additional FVIII dose is important from a health economics perspective. In clinical practice, FVIII concentrates are frequently chosen for these patients, but optimal dosing strategies remain uncertain.

Global coagulation assays, including clot waveform analysis (CWA), thrombin generation assays (TGAs), and rotational thromboelastometry (ROTEM), have emerged as useful tools for characterizing emicizumab-driven hemostasis and monitoring concomitant therapies [9,10,13]. We previously used these assays to demonstrate that emicizumab could improve coagulation potential in congenital and acquired HA, and to establish assay conditions for evaluating emicizumab in neonatal and pediatric settings [[14], [15], [16], [17]].

However, prospective data integrating global coagulation assays with real-world FVIII use in people with HA without inhibitors treated with emicizumab remain limited. Therefore, the multicenter “Coagulation potential of concomitant factor VIII administration in people with hemophilia A receiving emicizumab prophylaxis” (CAGUYAMA) study was designed to evaluate global coagulation potential, as assessed by prespecified functional assay parameters, immediately before FVIII administration (pre-FVIII) and after FVIII administration (post-FVIII) in people with HA receiving emicizumab prophylaxis, and to estimate an appropriate FVIII dosing regimen that balances efficacy and safety. The detailed study protocol has been published previously [18]. Here, we report the final clinical and laboratory findings of the CAGUYAMA study.

## 2 Methods

### 2.1 Study design and oversight

**Figure 1:**
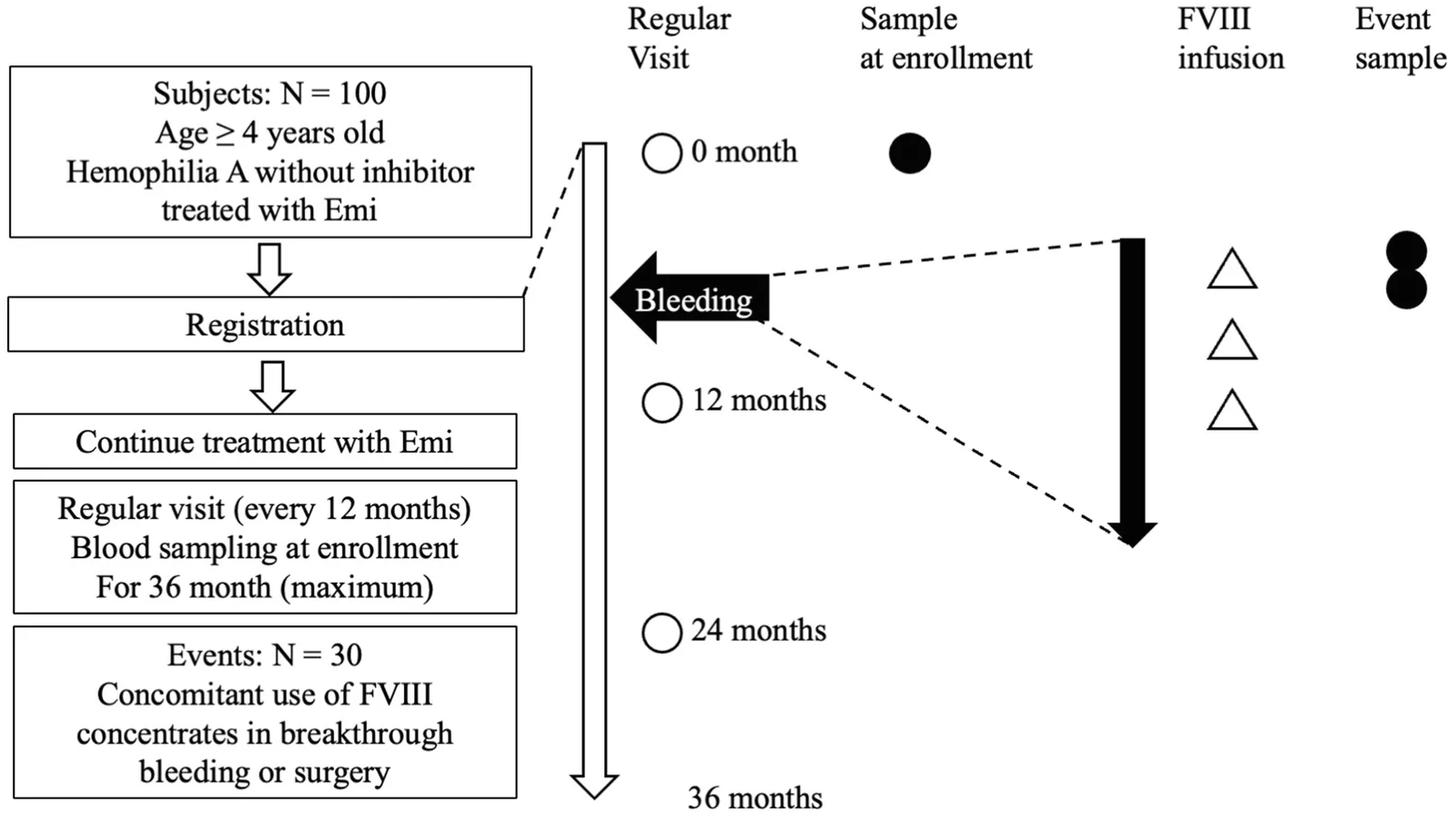
Study design and sampling schedule. The “Coagulation potential of concomitant factor VIII administration in people with hemophilia A receiving emicizumab prophylaxis” study design: people with hemophilia A without inhibitors receiving emicizumab (*N* = 100; age ≥ 4 years) were enrolled, continued emicizumab therapy, and attended regular visits every 12 months for up to 36 months. A baseline blood sample was obtained at enrollment. Factor (F)VIII-treated “events” (target *N* = 30) included breakthrough bleeding or surgeries requiring concomitant FVIII; event blood samples were collected at the time of FVIII infusion. Emi, emicizumab.

The CAGUYAMA study (jRCTs051210137) was a prospective, multicenter, open-label, nonrandomized clinical trial conducted at 13 hemophilia treatment centers in Japan. The study design, eligibility criteria, and planned analyses have been described in detail in the protocol article [18]. Briefly, the study comprised a 36-month observation period, during which enrolled people with HA receiving emicizumab prophylaxis were monitored for breakthrough bleeding and surgical events requiring additional FVIII supplementation (Figure 1).

The study was approved by the institutional review boards of all participating centers and was conducted in accordance with the Declaration of Helsinki and the International Council for Harmonization Good Clinical Practice guidelines. Written informed consent was obtained from all participants or their legal guardians prior to enrollment.

### 2.2 Participants

Male participants aged ≥4 years with congenital HA and no history of FVIII inhibitors who were receiving emicizumab prophylaxis according to local clinical practice were eligible for inclusion. Key inclusion criteria were severe or moderate HA, as defined by the International Society on Thrombosis and Haemostasis Scientific and Standardization Committee [19], stable emicizumab prophylaxis for at least 4 weeks prior to enrollment, and the ability to attend study visits and provide blood samples. Exclusion criteria included a current or past history of FVIII inhibitors, clinically significant hepatic dysfunction, uncontrolled thromboembolic or hypercoagulable conditions, or any other condition deemed unsuitable for study participation by the investigator.

### 2.3 Emicizumab prophylaxis and additional FVIII treatment during events

Emicizumab prophylaxis (loading and maintenance dose phases) was administered according to the prescribing information and local clinical practice; the dosing regimen was not prespecified in the protocol [18]. At the time of the breakthrough bleed or prior to invasive procedures, treating physicians administered FVIII concentrates at a protocol-specified initial dose of a target dose of 30 IU/kg (allowable range, 25–35 IU/kg) (standard or extended half-life formulations) in accordance with protocol recommendations. This dose was selected *a priori* for this exploratory study to examine whether a moderate single FVIII dose, superimposed on the baseline hemostatic contribution of emicizumab, could provide measurable additional coagulation potential and clinically adequate initial hemostasis. The protocol dose was not intended to replace individualized perioperative management or to define a sufficient dose for every major surgical procedure. Additional FVIII doses or other hemostatic therapies were permitted as clinically indicated and documented. An “event” was defined as any bleeding episode or surgical/invasive procedure for which FVIII was administered. Each event was analyzed independently, and a single patient could contribute to multiple events.

### 2.4 Blood sampling

For each event, venous blood samples were collected at 2 prespecified time points: pre-FVIII and 15 to 30 minutes post-FVIII. At enrollment, a separate baseline sample was obtained during a routine visit in the absence of recent FVIII exposure. All samples were centrifuged to prepare platelet-poor plasma and stored at −80 °C in a central laboratory until batch analysis. Global coagulation assays were analyzed centrally using prespecified assay conditions to minimize interlaboratory variability in this exploratory study.

### 2.5 Global coagulation assays

In this study, “global coagulation potential” refers to functional coagulation characteristics assessed under prespecified global assay conditions. This term was used to describe assay-based changes pre- and post-FVIII and was not intended to represent a validated clinical hemostatic threshold or a direct surrogate for measured FVIII activity.

### 2.6 CWA

**Figure 2:**
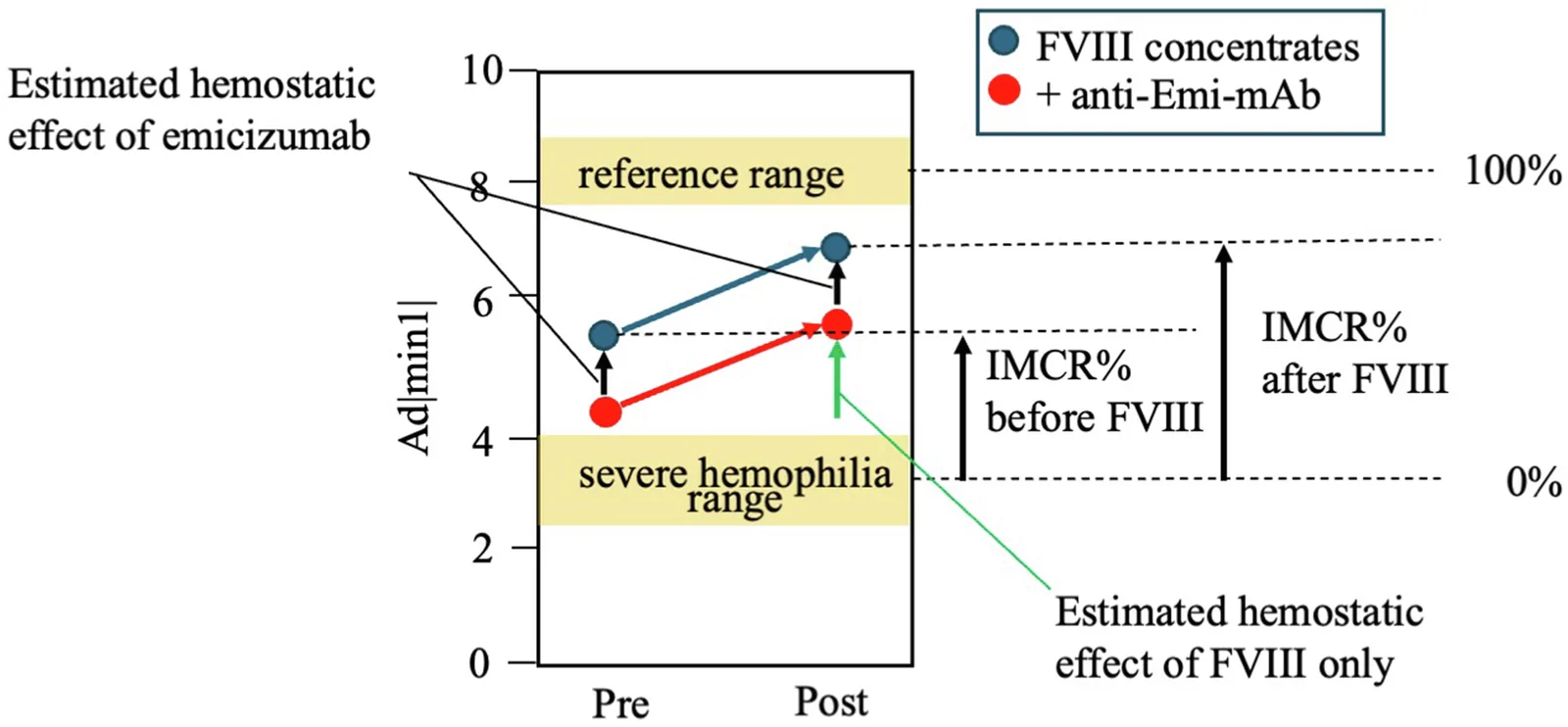
Adjusted clot waveform analysis was performed before factor (F)VIII administration (Pre) and after FVIII administration (Post) during emicizumab treatment, with the FVIII effect isolated using anti-emicizumab monoclonal antibodies (anti–Emi-mAbs). Global coagulation potential was assessed using adjusted clot waveform analysis. Absorbance changes were converted into coagulation curves. The first derivative of transmittance was used to determine the maximum coagulation rate (|min1|), which was then rescaled by redefining the postcoagulation minimum transmittance as 0% to yield the adjusted |min1| (Ad|min1|). To quantify improvement, the percentage improvement in |min1| (IMCR%) was calculated using pooled normal plasma (100%) and untreated severe hemophilia A plasma (0%) as references. Bars (or data points) show Pre- vs Post-FVIII values during emicizumab treatment and, where indicated, values for anti–Emi-mAbs to neutralize emicizumab activity and isolate the FVIII contribution alone. Shaded bands indicate the reference interval for normal and severe hemophilia A plasma.

Global coagulation potential was assessed using an adjusted CWA based on a mixture of prothrombin time (PT) and activated partial thromboplastin time (aPTT) reagents (PT:aPTT:buffer = 1:15:135 [v:v:v]), as previously described for use in the presence of emicizumab [9,14,17]. Coagulation was triggered by adding 25 mM CaCl_2_ to citrated plasma preincubated with the PT/aPTT mixture. Absorbance changes over time were converted to coagulation curves, and the first derivative of the optical density curve was used to calculate the maximum coagulation rate (|min1|). The minimum transmittance value in the postcoagulation phase was reset to 0%, and the adjusted |min1| (Ad|min1|) was used as the primary parameter. To quantify the relative improvement in global coagulation, the percentage improvement in |min1| (IMCR%) was calculated using reference values from normal pooled plasma (100%) and untreated severe HA plasma (0%; Figure 2). To differentiate the contribution of FVIII from that of emicizumab, selected samples were remeasured after incubation with anti-emicizumab monoclonal antibodies (anti–Emi-mAbs), which specifically and completely neutralize emicizumab activity without interfering with FVIII activity [20,21].

### 2.7 TGA

Thrombin generation was quantified using a modified, calibrated, automated thrombogram method optimized for emicizumab-containing plasma, employing low concentrations of recombinant tissue factor (TF; 0.5 pM), ellagic acid (0.3 μM), and phospholipid (PL) vesicles (4 μM), as previously reported [13]. Thrombin generation was initiated by adding the TF/ellagic acid/PL mixture and was continuously monitored using a fluorogenic substrate (Z-Gly-Gly-Arg-AMC, Bachem). Peak thrombin concentration (Peak-Th) was the primary endpoint. The IMCR% for Peak-Th was calculated in a manner analogous to CWA, using normal pooled plasma (100%) and untreated severe HA plasma (0%) as references. When indicated, anti–Emi-mAbs were added to selectively neutralize emicizumab activity and isolate the FVIII-dependent component.

### 2.8 ROTEM

Whole blood clot formation was evaluated using nonactivated ROTEM (nonactivated thromboelastometry mode), which is sensitive to hemostatic changes driven by emicizumab [10,22,23]. Clotting time (CT) was used as the primary ROTEM parameter. For selected events, ROTEM measurements were repeated following incubation with anti–Emi-mAbs to assess FVIII-dependent CT shortening.

### 2.9 Clinical efficacy and safety assessments

The clinical hemostatic efficacy of each event was evaluated by local investigators using prespecified categorical criteria (excellent, good, moderate, or poor) based on bleeding control, the need for additional hemostatic treatment, and perioperative outcomes. For analytical purposes, hemostasis was considered effective when rated as excellent or good.

Adverse events (AEs), AEs of special interest (AESIs; such as systemic hypersensitivity reactions, thromboembolic events, or thrombotic microangiopathy), and serious AEs were recorded throughout the observation period. Routine laboratory parameters, including platelet counts, and markers of coagulation and fibrinolysis, were monitored at scheduled visits and during FVIII-treated events.

### 2.10 Endpoints

The *primary endpoint* was defined as the change in Ad|min1|-derived IMCR% between the pre- and post-FVIII time points in the all-events analysis set (Figure 2).

*Secondary endpoints* included:

• Clinical hemostatic efficacy for each FVIII-treated event
• Changes in Peak-Th IMCR% in the TGA between pre- and post-FVIII
• Changes in ROTEM CT between pre- and post-FVIII
• Comparison of CWA and TGA IMCR% values before and after the neutralizing emicizumab activity of anti–Emi-mAbs.
• Incidence of AEs and AESIs

*Exploratory endpoints* included:

• Correlations between plasma emicizumab concentrations and global coagulation assay parameters at enrollment.
• Associations among FVIII dose, FVIII product type (standard or extended half-life), and changes in global coagulation parameters.

### 2.11 Statistical analysis

Continuous variables are presented as means ± SDs or medians (ranges), as appropriate, while categorical variables are expressed as counts and percentages. Paired comparisons between pre- and post-FVIII parameters were performed using paired *t*-tests or Wilcoxon signed-rank tests, depending on data distribution. Correlations were evaluated using Pearson or Spearman correlation coefficients, as appropriate. A 2-sided *P* value < .05 was considered statistically significant. The statistical analyses were performed using SAS software (version 9.4; SAS Institute Inc).

## 3 Results

### 3.1 Patient characteristics and FVIII-treated events

A total of 100 people with HA without inhibitors who were on emicizumab prophylaxis were enrolled across 13 participating centers in Japan. The median age at enrollment was 31 years (range, 4-73), and the median duration of prior emicizumab exposure was 30 months (range, 0-66). During the 36-month observation period, 32 FVIII-treated events were reported in 24 participants (22 with severe types and 2 with moderate types). The median age at the time of the events was 35 years (range, 4-73). Fifteen events (46.9%) were breakthrough bleeds, and 17 (53.1%) were surgical or invasive procedures (Table). Event types included joint, muscle, nasal, gastrointestinal, and subcutaneous bleeds, as well as procedures such as tooth extraction, synovectomy, total knee arthroplasty, and endoscopic polypectomy. Fourteen events (43.8%) were treated with standard half-life FVIII, and 18 (56.2%) with extended half-life FVIII. The mean ± SD FVIII dose was 30.8 ± 3.3 U/kg, resulting in a mean ± SD postinfusion FVIII activity of 77.1% ± 20.4% and an *in vivo* recovery of 2.5% ± 0.7% U/kg, which is consistent with previously reported recovery rates in people with HA without inhibitors [24,25].

**Table:** Event types and details in the “Coagulation potential of concomitant factor VIII administration in people with hemophilia A receiving emicizumab prophylaxis” study.

Event type	Details
Breakthrough bleeding (15 events in 11 patients)	Joint (*n* = 7), muscle (*n* = 2), nasal (*n* = 2), gastrointestinal bleeding (*n* = 2), fracture (*n* = 1), and subcutaneous (*n* = 1)
Surgical procedure (17 events in 13 patients)	Tooth extraction (*n* = 6), synovectomy (*n* = 3), total knee arthroplasty (*n* = 2), arthrocentesis (*n* = 1), prostate biopsy (*n* = 1), subcutaneous mass excision (*n* = 1), suture removal after tumor excision (*n* = 1), buccal mucosa mucocele excision (*n* = 1), and endoscopic colon polypectomy (*n* = 1)

### 3.2 Primary endpoint: IMCR% in the CWA

**Figure 3:**
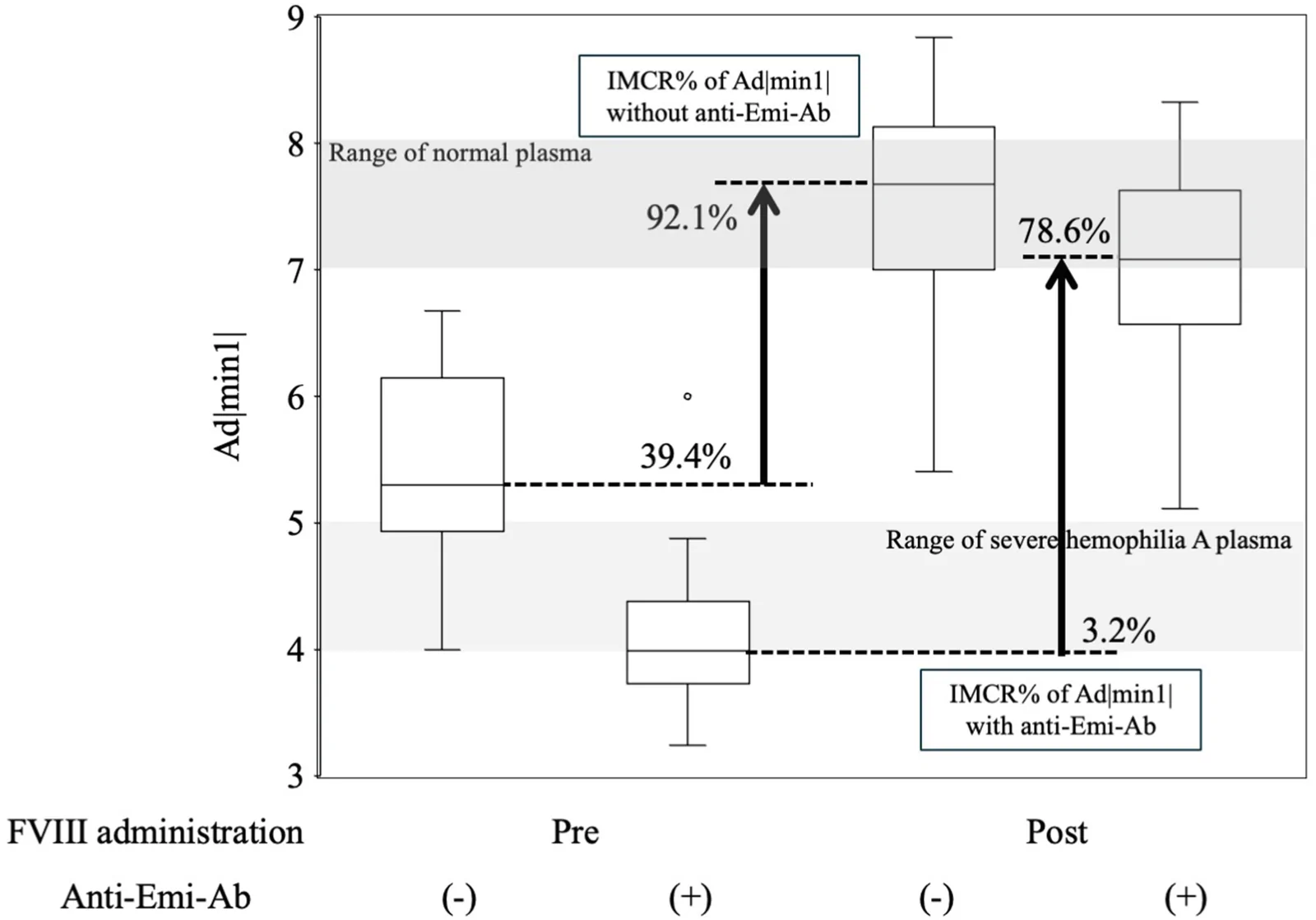
Clot waveform analysis was performed before (F)VIII administration (Pre) and after FVIII administration (Post), with and without the addition of anti-emicizumab monoclonal antibodies (anti–Emi-mAbs). Adjusted clot waveform analysis was performed on citrated plasma collected from patients Pre- and Post-FVIII. To isolate the FVIII contribution from that of emicizumab, aliquots of the same samples were reassessed after incubation with anti–Emi-mAbs, which neutralize emicizumab activity without affecting FVIII activity. The graph shows the percentage improvement in the maximum coagulation rate (IMCR%) relative to pooled normal plasma (100%) and untreated severe hemophilia A plasma (0%); shaded bands indicate the normal and severe reference intervals. Ad|min1|, adjusted maximum coagulation rate.

In the all-events analysis set, the median Ad|min1|-derived IMCR% increased markedly from 39.4% pre-FVIII to 92.1% post-FVIII (Figure 3). The normal reference level was defined as 100% relative to pooled normal plasma, and the severe HA reference level as 0% relative to untreated baseline samples. Most post-FVIII values approached or reached the normal range, indicating near-normalization of global coagulation under the assay conditions used in this study following administration of the protocol-specified initial FVIII dose of a target dose of 30 IU/kg (allowable range, 25–35 IU/kg). After incubation with anti–Emi-mAbs, the IMCR% values decreased to 3.2% pre-FVIII and 78.6% post-FVIII (Figure 3). Based on these results, the calculated FVIII-mediated improvement was 52.7% in the presence of emicizumab and 75.4% after emicizumab activity was neutralized, indicating that emicizumab contributes to the baseline assay-based coagulation potential and that FVIII provides an additional functional effect. Across all events, Ad|min1| showed a moderate positive correlation with plasma emicizumab concentration (Spearman’s ρ = .47; *P* < .0001). This association disappeared after neutralizing emicizumab activity (ρ = .12; *P* = .23), corroborating the assay’s specificity for emicizumab-dependent activity.

### 3.3 Secondary endpoints

#### 3.3.1 IMCR% in the TGA

**Figure 4:**
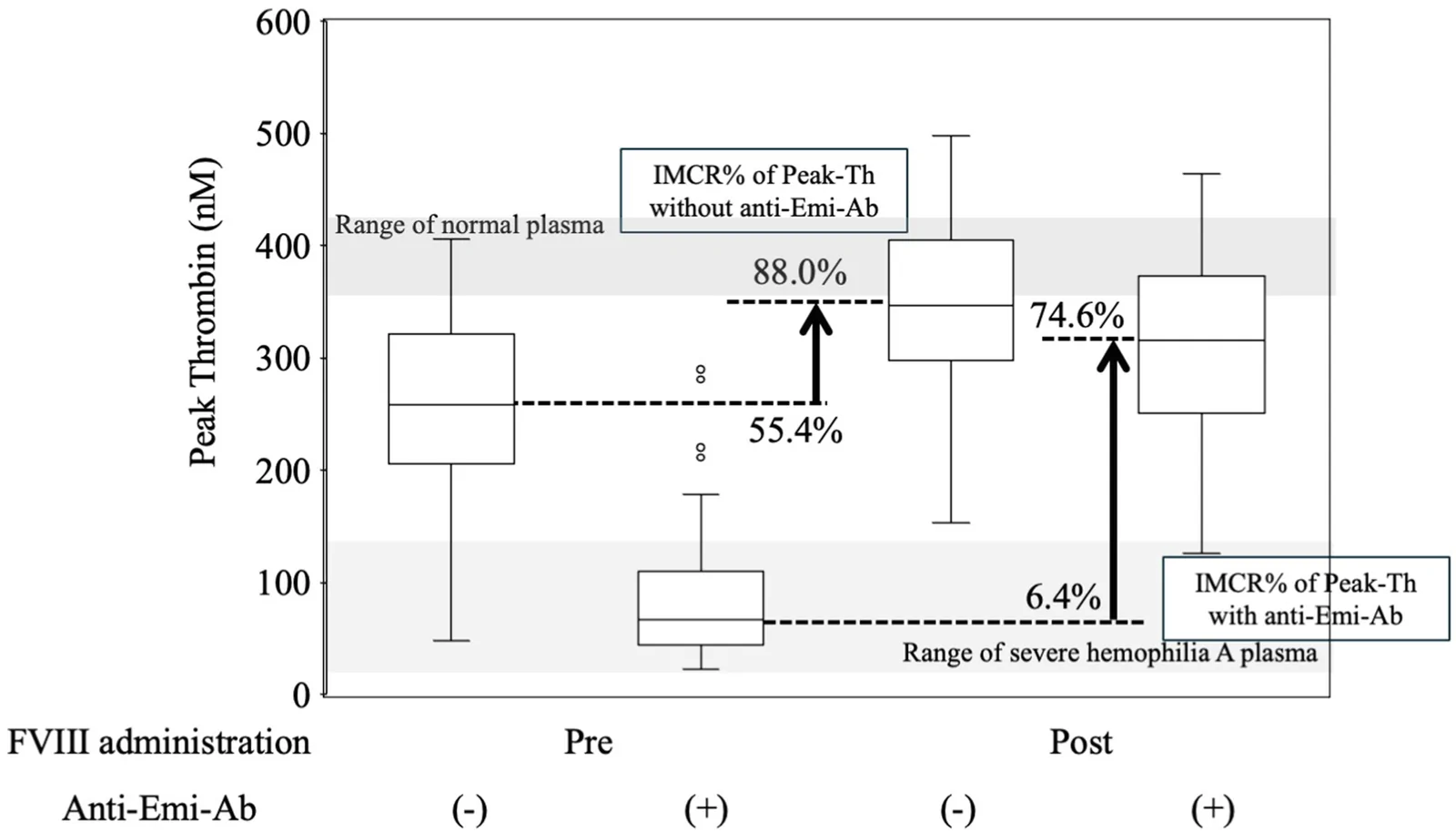
Thrombin generation assay before factor (F)VIII administration (Pre) and after FVIII administration (Post), with and without anti-emicizumab monoclonal antibodies (anti–Emi-mAbs). The thrombin generation assay was performed on citrated plasma obtained Pre- and Post-FVIII. The primary readouts were time to peak (minutes) and peak thrombin (Peak-Th; nM). To isolate FVIII-specific effects, paired aliquots were reassessed after incubation with anti–Emi-mAbs. IMCR%, percentage improvement in the maximum coagulation rate (applied to thrombin generation assay) relative to reference plasmas.

In the TGA triggered by the TF/ellagic acid/PL mixture, median Peak-Th values increased from 249 nM pre-FVIII to 348 nM post-FVIII, corresponding to IMCR% values of 55.4% and 88.0%, respectively (Figure 4). In the presence of anti–Emi-mAbs, IMCR% values were 6.4% pre-FVIII and 74.6% post-FVIII, resulting in calculated FVIII effects of 32.6% under emicizumab prophylaxis and 68.2% after neutralizing emicizumab activity. These findings indicate that the TGA captures FVIII-mediated additive effects but is less sensitive than CWA to baseline emicizumab activity, consistent with prior mechanistic observations [13]. Previous studies have also demonstrated that the procoagulant activity of emicizumab is only modestly reflected in the TGA, whereas CWA, particularly with mixed PT/aPTT triggers, exhibits greater analytical sensitivity [9,13]. Exploratory correlation analyses showed that emicizumab concentration positively correlated with Peak-Th (mean ± SD, 249 ± 84.5 nM pre-FVIII vs 348 ± 90.3 nM post-FVIII; ρ = .42; *P* < .0001) and lag time (mean ± SD, 9.8 ± 2.3 minutes pre-FVIII vs 7.1 ± 1.6 minutes post-FVIII; ρ = .43; *P* < .0001), and inversely with time to peak (mean ± SD, 16.4 ± 3.2 minutes pre-FVIII vs 12.3 ± 2.7 minutes post-FVIII; ρ = −.46; *P* < .0001). These relationships were attenuated after neutralizing emicizumab activity (Peak-Th: ρ = .21, *P* = .03; lag time: ρ = .26, *P* = .009; time to peak: ρ = −.29, *P* = .003), confirming TGA’s specificity for emicizumab-dependent coagulation activity.

#### 3.3.2 CT in ROTEM

**Figure 5:**
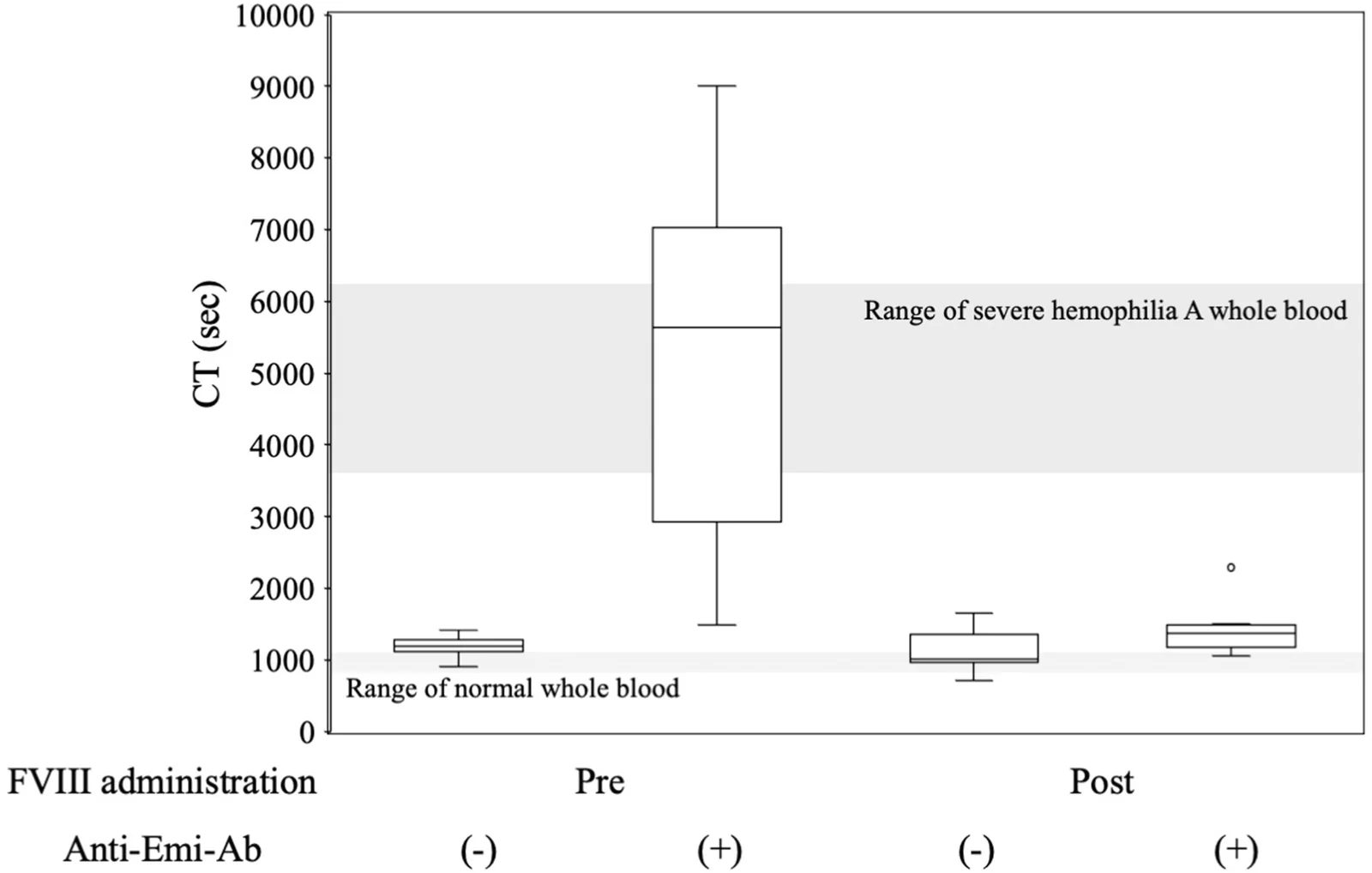
Rotational thromboelastometry before factor (F)VIII administration (Pre) and after FVIII administration (Post), with and without anti-emicizumab monoclonal antibodies (anti–Emi-mAbs). Whole blood rotational thromboelastometry (nonactivated thromboelastometry mode) was performed Pre- and Post-FVIII. The primary parameter was clotting time (CT; seconds; clot formation time is shown where available). Corresponding sample aliquots were additionally assessed after incubation with anti–Emi-mAbs to evaluate the FVIII-specific component in the presence of emicizumab.

ROTEM data were available for 11 evaluable events. In the nonactivated thromboelastometry mode, the mean ± SD CT decreased slightly from 1190 ± 169 seconds pre-FVIII to 1158 ± 301 seconds post-FVIII, remaining above the normal reference range (mean ± SD, 938 ± 128 seconds). In contrast, when assessed after incubation with anti–Emi-mAbs, the mean ± SD CT decreased markedly from 5213 ± 2915 seconds to 1459 ± 447 seconds, confirming a pronounced FVIII-dependent effect (Figure 5). Clot formation time and maximum clot firmness did not change significantly (mean ± SD clot formation time: 187 ± 56 seconds pre-FVIII vs 179 ± 52 seconds post-FVIII; maximum clot firmness: 51.6 ± 5.2 mm vs 52.1 ± 4.8 mm). These results suggest that ROTEM primarily reflects the dynamics of the coagulation initiation phase in this setting. Correlation analysis revealed a negative association between CT and plasma emicizumab concentration (ρ = −.41; *P* = .007), which disappeared after neutralizing emicizumab activity (ρ = −.12; *P* = .23), indicating assay specificity. These results align with prior observations that ROTEM is particularly informative for evaluating the combined use of emicizumab with FVIII or bypassing agents [[14], [15], [16], [17]].

### 3.4 Exploratory analyses

At enrollment, the mean ± SD plasma emicizumab concentration was 51.2 ± 17.8 μg/mL. A moderate positive correlation was observed between emicizumab concentration and baseline Ad|min1| in CWA (r = .42), whereas the correlation with Peak-Th in the TGA was minimal (r = .05). These findings reinforced the notion that CWA adjusted with PT/aPTT reagents is particularly sensitive to the procoagulant activity of emicizumab [9,14,17]. No significant association was identified between FVIII product type (standard vs extended half-life) and changes in parameters measured by the global assays, although the study was not powered to detect such differences.

### 3.5 Clinical hemostatic efficacy

All 32 events treated with FVIII achieved clinical hemostatic efficacy. Representative event categories included 12 joint or muscle bleeds, 2 gastrointestinal bleeds, 6 dental procedures, and 5 orthopedic surgeries. For breakthrough bleeding, bleeding was controlled without the need for rescue therapy, and no unexpected rebleeding occurred. For surgical procedures, the perioperative course was uneventful, without excessive bleeding or an unplanned need for additional hemostatic interventions.

### 3.6 Safety

Five patients experienced 5 AEs during the study (febrile seizure, COVID-19 infection, femoral shaft fracture, femoral neck fracture, and intra-abdominal hemorrhage), none of which were deemed related to the administration of emicizumab or FVIII. No systemic hypersensitivity reactions, thromboembolic events, or thrombotic microangiopathy were observed. Platelet counts and routine coagulation or fibrinolysis parameters remained stable, with no clinically relevant changes throughout the study.

## 4 Discussion

In this prospective, multicenter clinical study of people with HA without inhibitors treated with emicizumab, we demonstrated that FVIII supplementation at a target dose of 30 IU/kg (allowable range, 25–35 IU/kg) was associated with effective hemostasis across the evaluated breakthrough bleeding and surgical events, with no observed thromboembolic events or thrombotic microangiopathy. Global coagulation assays revealed an additive FVIII-mediated increase in global coagulation potential beyond the baseline hemostatic activity associated with emicizumab. Given the exploratory, nonrandomized design, and the limited number of evaluable events, these findings support the feasibility of the initial dosing approach but do not establish a target dose of 30 IU/kg (allowable range, 25–35 IU/kg) as the optimal dose for all bleeding or surgical situations, particularly major surgery. Consistent with previous reports [9,11], emicizumab improved the global coagulation potential from a severe to a mild hemophilia phenotype, corresponding to an FVIII-equivalent activity of approximately 10% to 20%. In CWA, baseline IMCR% values (∼40%) with emicizumab prophylaxis corresponded to this mild phenotype, whereas FVIII supplementation (a target dose of 30 IU/kg (allowable range, 25–35 IU/kg) increased the IMCR% to >90%, indicating near-normalization of coagulation potential under the assay conditions used in this study. Similar, albeit less pronounced, trends were observed in the TGA results. Neutralizing emicizumab activity with anti–Emi-mAbs enabled the separation of the individual functional contributions of FVIII and emicizumab. As expected, the FVIII-induced improvement was greater in the absence of emicizumab; however, it remained substantial even in the presence of emicizumab, indicating an additive functional effect of FVIII in emicizumab-containing samples. These findings align with previous mechanistic and *ex vivo* studies demonstrating that concomitant FVIII further enhances emicizumab-associated thrombin generation in FVIII-deficient plasma models [13,15,26].

Real-world and registry data suggest that standard FVIII dosing can be effective for hemostasis during breakthrough bleeding and surgeries in people with HA without inhibitors on emicizumab prophylaxis. However, dosing strategies vary, and optimal titration to avoid under- or overtreatment remains a clinical challenge [27]. In the present study, the a target dose of 30 IU/kg (allowable range, 25–35 IU/kg) dose substantially improved global coagulation parameters and was associated with effective clinical hemostasis, without any observed thrombotic complications. Nevertheless, because this study did not include a comparator dosing strategy and was not designed to evaluate dose optimization, these findings should be interpreted as supporting the feasibility of the initial dosing approach rather than establishing its superiority, equivalence to conventional dosing, or suitability for all clinical situations. ROTEM demonstrated only a modest decrease in CT while emicizumab was active, but showed a pronounced FVIII effect after emicizumab activity was neutralized. Together with a previous report [10], this suggests that ROTEM may provide complementary information for assessing the combined activity of emicizumab and FVIII, particularly in high-risk or perioperative settings. However, because ROTEM data were available for only a limited number of evaluable events and the apparent sensitivity to FVIII-related changes was lower in the presence of active emicizumab, the role of ROTEM in guiding perioperative FVIII dosing requires further investigation. Interpretation of the FVIII dose used for major surgery also requires caution. In our study, 2 patients underwent major orthopedic procedures (including a total knee arthroplasty) with FVIII doses slightly exceeding a target dose of 30 IU/kg (allowable range, 25–35 IU/kg), achieving uneventful hemostasis without thrombotic complications. However, because the number of surgical cases was very limited and no comparator dosing strategy was included, these observations do not establish that an initial FVIII dose of approximately 30 U/kg is sufficient or optimal for major surgeries in people with HA without inhibitors treated with emicizumab. Rather, perioperative FVIII management for major surgery should remain individualized according to the type of procedure, anticipated bleeding risk, measured FVIII activity where appropriate, clinical response, and the need for additional postoperative dosing. Further prospective studies are needed to refine FVIII dose selection in the context of emicizumab prophylaxis, considering efficacy, safety, and potential cost-effectiveness.

A notable finding from our exploratory analyses was the moderate correlation between plasma emicizumab concentration and Ad|min1| in CWA, in contrast to the weaker association with Peak-Th in the TGA. Additionally, the magnitude of FVIII-induced improvements in CWA and the TGA varied among patients, reflecting interindividual variability in FVIII responsiveness. These differences did not correlate with age, baseline FVIII activity, or emicizumab trough levels, possibly indicating the influence of other patient-specific factors. This finding aligns with previous work showing that an adjusted PT/aPTT-triggered CWA is highly responsive to the effects of emicizumab and can differentiate between drug doses and developmental hemostatic states [9,14,17]. Taken together, the global assays appear complementary but not interchangeable; even in the TGA, parameter selection was important, as conversions based on endogenous thrombin potential vs Peak-Th yielded different estimates, highlighting parameter dependence. In contrast, the TGA may offer broader insights into the thrombin-generation potential across various triggers. Although head-to-head comparisons are limited, their use alongside CWA has been advocated in mechanistic studies and clinical evaluations. Our results support a complementary role for these assays: CWA is advantageous for quantifying baseline emicizumab-driven activity, while TGA provides an integrated assessment of the cumulative impact of additional hemostatic agents. ROTEM may provide complementary whole-blood information in high-risk settings, such as during surgery or in cases of suspected hypercoagulability. However, its results are not directly comparable to those of plasma-based assays and require assay- and parameter-specific validation, particularly in the presence of emicizumab.

Although adjusted CWA can, in principle, be performed using optical clot-detection data generated by compatible coagulation analyzers, its routine clinical implementation remains limited in Japan, particularly outside specialized centers. In addition, trigger reagents and analytical conditions for adjusted CWA in the presence of emicizumab have not yet been standardized across laboratories; these conditions may differ between institutions and can influence assay results. For these reasons, the present study employed prespecified trigger conditions and centralized batch analysis to ensure analytical consistency. Before adjusted CWA can be routinely used to inform FVIII dosing during emicizumab prophylaxis, further prospective validation is required regarding interlaboratory reproducibility, locally applicable reference ranges, quality-control procedures, and standardized reporting formats. Until such standardization is established, CWA should be regarded as a potentially informative adjunct to clinical assessment and conventional laboratory monitoring, rather than a stand-alone tool for determining FVIII dosing.

Clinically, the complete hemostatic success across various bleeding and surgical events is reassuring. The absence of thrombotic or microangiopathic events, despite the concomitant use of emicizumab and FVIII, was consistent with long-term follow-up data from people with HA without inhibitors receiving emicizumab prophylaxis, which reported continued safety and favorable outcomes during routine and perioperative care [28,29], as well as with the Nuwiq for perioperative management of patients with Haemophilia A on emicizumab regular prophylaxis protocol study, which specifically addresses the perioperative use of FVIII with emicizumab treatment [30]. Notably, in the present study, no AESIs, including thrombotic events, and thrombotic microangiopathy, were observed among the 32 events treated with FVIII. Although the number of treated events was insufficient to assess uncommon safety outcomes, these observations provide supportive prospective safety information regarding concomitant FVIII use during emicizumab prophylaxis. Our results also have implications for other populations treated with emicizumab. Studies on acquired HA (the phase III study of emicizumab in patients with acquired hemophilia A [AGEHA] study and related research) [31] and on neonates/infants with HA [14,17] have highlighted the need for careful integration of emicizumab with conventional hemostatic agents across populations with varying baseline coagulation profiles. The CAGUYAMA study extends this evidence to people with HA without inhibitors, providing a basis for further evaluation of assay-informed FVIII dosing strategies.

This study has several limitations. First, only 32 FVIII-treated events in 24 participants were evaluable; therefore, uncommon AEs and event-specific outcomes, particularly those related to major surgery, could not be reliably characterized. Second, FVIII dosing was not randomized, and no comparator dosing strategy was included. Consequently, this study could not determine whether the protocol-specified initial dose of a target dose of 30 IU/kg (allowable range, 25–35 IU/kg) is optimal, equivalent to conventional dosing, or sufficient across heterogeneous bleeding and surgical risks. Third, additional FVIII administration was allowed at the clinician’s discretion, which may have influenced clinical outcomes and limited the interpretation of the initial dose alone. Fourth, the study population and clinical events were derived from Japanese centers and may not fully reflect clinical practice patterns or patient characteristics in other countries. Finally, adjusted CWA was performed under prespecified assay conditions with centralized analysis; therefore, the reproducibility and clinically applicable decision thresholds required for implementation outside specialized laboratories were not evaluated.

In conclusion, in this prospective exploratory study, FVIII supplementation at a target dose of 30 IU/kg (allowable range, 25–35 IU/kg) was associated with effective hemostasis and no thrombotic complications across the evaluated breakthrough bleeding and surgical events in people with HA without inhibitors treated with emicizumab. Global coagulation assays revealed substantial additive effects of FVIII beyond the baseline activity of emicizumab, with adjusted CWA being particularly sensitive to emicizumab-associated coagulation changes. These findings support further evaluation of a moderate initial FVIII dosing strategy and assay-informed management during emicizumab prophylaxis. However, larger comparative studies are required before standardized dosing recommendations, particularly for major surgery, can be established.
